# Using Co-Designed Norm-Based Nudges to Promote Mental Health Help-Seeking Intentions Among Older Adults: Protocol for a Randomized Controlled Trial

**DOI:** 10.2196/103455

**Published:** 2026-07-21

**Authors:** Tianyin Liu, Ken Ho Kan Liu, Dara Kiu Yi Leung, Wen Zhang, Wai Wai Kwok, Gloria Hoi Yan Wong, Terry Yat Sang Lum

**Affiliations:** 1 Department of Applied Social Sciences, Hong Kong Polytechnic University, 11 Yuk Choi Rd, Hung Hom, Hong Kong, 999077, China (Hong Kong), 852 27665015; 2 Department of Social Work, Chinese University of Hong Kong, Hong Kong, China (Hong Kong); 3 School of Nursing and Health Sciences, Hong Kong Metropolitan University, Hong Kong, China (Hong Kong); 4 Department of Social Work and Social Administration, University of Hong Kong, Hong Kong, China (Hong Kong); 5 School of Psychology and Clinical Language Sciences, University of Reading, Reading, England, United Kingdom

**Keywords:** nudge, social norm, help-seeking, mental health, older adults, randomized controlled trial, texting

## Abstract

**Background:**

Older adults face a substantial burden of common mental disorders, yet they are less likely to seek professional help compared to younger adults. In Chinese contexts, collectivist values and stigma may further deter help-seeking. Social norms are central to the theory of planned behavior (TPB) and nudge theory, suggesting that norm-based messages, especially those that are visually engaging, could strengthen perceived social approval and subsequent help-seeking intentions.

**Objective:**

This study aims to evaluate the effectiveness of co-designed norm-based nudges, delivered in descriptive (descriptive norm nudge) or pictorial forms (pictorial norm nudge), in improving subjective norms and intentions to seek mental health help among older adults, compared to an information-only control.

**Methods:**

This 3-arm randomized controlled trial (1:1:1) recruits adults aged ≥60 years via community partners in Hong Kong. Participants receive daily texting for 14 days and are allocated to the pictorial norm nudge (descriptive text combined with pictorial cultural games that evoke social support), the descriptive norm nudge (descriptive text only), or the control (mental health service information) groups. Assessments are conducted at baseline (T0), after intervention (T1), and at a 3-month follow-up (T2). Primary outcomes are subjective norms and help-seeking intentions, measured using the Chinese version of the TPB questionnaire. Secondary outcomes include perceived behavioral control, help-seeking attitudes, perceived barriers, depression, anxiety, and loneliness. A 2-level linear mixed model will be used in intention-to-treat analyses to examine the intervention’s effectiveness.

**Results:**

The study grant was awarded in June 2023, with recruitment of participants to the randomized controlled trial starting in December 2024 and concluding in June 2026. As of June 30, 2026, a total of 532 eligible participants had completed the baseline assessment. Among them, 470 (88.34%) completed the postintervention, and 408 (76.69%) completed the 3-month follow-up assessment assessments. Data collection and follow-ups are ongoing. A full report of the findings is expected by October 2026, with publication anticipated in early 2027.

**Conclusions:**

This study will provide critical insights into the feasibility and effectiveness of brief, co-designed nudges for promoting mental health help-seeking intentions. If effective, this approach will offer a scalable, culturally adaptive, and low-intensity strategy to improve mental health service use among older adults.

## Introduction

### Background

Mental health issues among older adults are an escalating global public health priority. Recent systematic reviews estimate that 15% to 20% of older adults experience major depressive disorder [1], while the global prevalence of depressive symptoms in this demographic reaches approximately 35% [2]. Early prevention and timely intervention are critical to reducing the impact of mental health conditions on individuals, reducing caregiver burden, and preventing the escalation of mild conditions into chronic psychiatric disorders or suicide [3-5]. Despite the availability of social services, older adults frequently delay or forgo professional help. This untreated duration often leads to exacerbated health outcomes and significantly higher longitudinal health care expenditures [6].

This underuse of mental health interventions is particularly pronounced among Chinese populations. Barriers include a strong cultural emphasis on self-reliance, reliance on informal or traditional resources, and a low perceived need for formal psychiatric help [7]. In high-density urban environments such as those in Hong Kong, despite a well-developed social service infrastructure, there remains a critical need for innovative strategies to bridge the gap between service availability and community uptake [8].

The theory of planned behavior (TPB) provides a robust framework for understanding the mechanisms underlying help-seeking, conceptualizing help-seeking as a complex, multistage process driven by attitudes, subjective norms, and perceived behavioral control [9,10]. In the context of mental health, this process is further complicated by social stigma, which is negatively correlated with help-seeking intentions among older adults with depression [11]. In older Chinese populations, cultural values such as “face-saving” and familial reliance significantly deter help-seeking [12-14]. Recognizing these nuances, a revised TPB model for Chinese contexts suggests that subjective norms (perceived social pressure and approval) serve as the primary antecedent influencing both attitudes and perceived behavioral control, ultimately driving intention [15]. Consequently, interventions targeting subjective norms may be the most effective means of achieving behavioral change.

“Nudging” alters choice architecture to steer individuals toward better decisions without restricting options [16]. Although it has been applied to geriatric health behaviors such as nutrition and vaccination [17], its application to mental health help-seeking remains nascent [18]. Existing interventions often rely on institutional-level gatekeeper training or psychoeducation [19,20]. However, daily, individual-level digital interventions offer a scalable alternative. For older Chinese adults, who are highly sensitive to social conformity, “social norm nudges” highlighting socially approved behaviors are hypothesized to be particularly effective [21,22]. Furthermore, incorporating visual cues (pictorial nudges) can improve attention, comprehension, and information recall, especially among populations with growing digital literacy [23-25]. To ensure cultural resonance, these nudges must be tailored through co-design with the target population.

### Objectives

This study aims to evaluate the effectiveness of descriptive norm nudges and pictorial norm nudges compared to an active control (mental health service information) in improving subjective norms and help-seeking intentions among older adults in Hong Kong. We hypothesize that both the descriptive norm nudges and the pictorial norm nudges groups will outperform the control group at postintervention assessment and at the 3-month follow-up.

## Methods

### Ethical Considerations

Ethics approval was granted by The Hong Kong Polytechnic University Institutional Review Board (reference HSEARS 20231109001; approval date: Nov 10, 2023). Participants receive compensation for their participation at the baseline, after the intervention, and 3 months after intervention. Written informed consent is obtained from all participants. Consent has been obtained for the use of any image-based nudge materials in which identifiable individuals appear. Model consent forms are available on request. Participants may withdraw from the study at any time. Confidentiality will be maintained through the use of unique participant codes, and study records will be accessible only to authorized research personnel. Findings will be disseminated via academic conferences and peer-reviewed publications.

### Study Design

This is a 3-arm, parallel-group randomized controlled trial (RCT) with a 1:1:1 allocation ratio. Participants complete assessments at 3 time points: baseline (T0), immediately after intervention (T1), and 3 months after intervention (T2). This protocol is reported in accordance with the SPIRIT (Standard Protocol Items: Recommendations for Interventional Trials) 2025 guidelines [26] (Checklist 1).

### Study Setting and Participants

The study is conducted in Hong Kong. Participants are recruited through the university research center website, social media, and community-based aged care services. The inclusion criteria are as follows: (1) aged ≥60 years, (2) able to read Chinese, and (3) ownership of a smartphone capable of receiving WhatsApp (Meta Platforms) texting (words and images). The exclusion criteria include the following: (1) a severe mental disorder, (2) cognitive impairment, (3) severe visual impairment, and (4) communication difficulties precluding informed consent.

### Intervention Program

#### Overview

The intervention was developed through a participatory co-design process. Specifically, 12 older adults, 2 researchers, and 1 graphic designer participated in 8 structured workshops guided by the design thinking principles of empathize, define, ideate, and prototype. The co-design process generated a set of reminiscence-oriented, metaphor-based hybrid nudges that combine short descriptive norm messages with culturally meaningful visuals (eg, childhood games) to normalize help-seeking and reduce psychological resistance. The nudges are designed to influence intention through several complementary mechanisms, including social norm activation, affective priming, cognitive simplification, action cues, and repeated exposure. Details of the behavioral mechanisms and examples of pictorial norm nudges and descriptive norm nudges are summarized in Table 1.

**Table 1.** Examples of pictorial norm nudges, descriptive norm nudges, and corresponding nudging strategies.

Strategies	Childhood games used	Descriptive norm nudges	Pictorial norm nudges
Action cue	Hopscotch	“Just take the first step, and you can go wherever you want.”	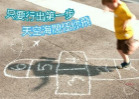
Positive frame	Slide	“Whew, it’s comfortable to let it out.”	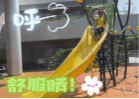
Peer influence	Seesaw	“No matter how many ups and downs you go through, as long as you have company, there is joy.”	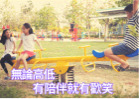

#### Pictorial Norm Nudge

Participants allocated to the pictorial norm nudge arm receive multimedia messages that combine descriptive norm-based text with pictorial stimuli. For example, a message might feature a hopscotch image encouraging the users to take the “first step,” using visuals that evoke autobiographical memories of peer support and resilience.

#### Descriptive Norm Nudge

In contrast, participants in the descriptive norm nudge arm receive text-only versions of the pictorial norm nudge messages. The wording is identical to that in the pictorial norm nudge arm, thereby isolating the added behavioral effect of visual cues.

#### Active Control

Participants in the active control group receive informational messages providing practical details about accessing local mental health and social care services, entirely devoid of normative framing or metaphors.

### Delivery Format and Participant Engagement

All participants receive 1 brief message daily for 14 consecutive days, requiring less than 30 seconds to read. To encourage engagement, all participants are invited to provide brief responses (eg, emoji reactions or short replies) following each message. Participants will be discontinued from the intervention if they demonstrate sustained nonengagement, defined as failing to open or respond to study messages for 3 consecutive days. Participants may also withdraw voluntarily at any time without penalty.

### Random Allocation and Blinding

Following the baseline assessment, a computer-generated sequence assigns participants to the pictorial norm nudge, descriptive norm nudge, or control groups in a 1:1:1 ratio. An independent staff member manages allocation concealment. Due to the visible nature of the intervention formats (pictorial messages, text-only messages, or informational content), participant blinding is limited. To minimize expectation and self-report bias, all groups receive interventions framed as mental health promotion messages, and identical assessment procedures are applied across groups. We use standardized and validated psychometric scales that do not directly reference the intervention materials. Although participant blinding is limited, the outcome assessors and the data analysis team will remain fully blinded to group allocation until all primary analyses are completed.

### Procedure

**Figure 1.**
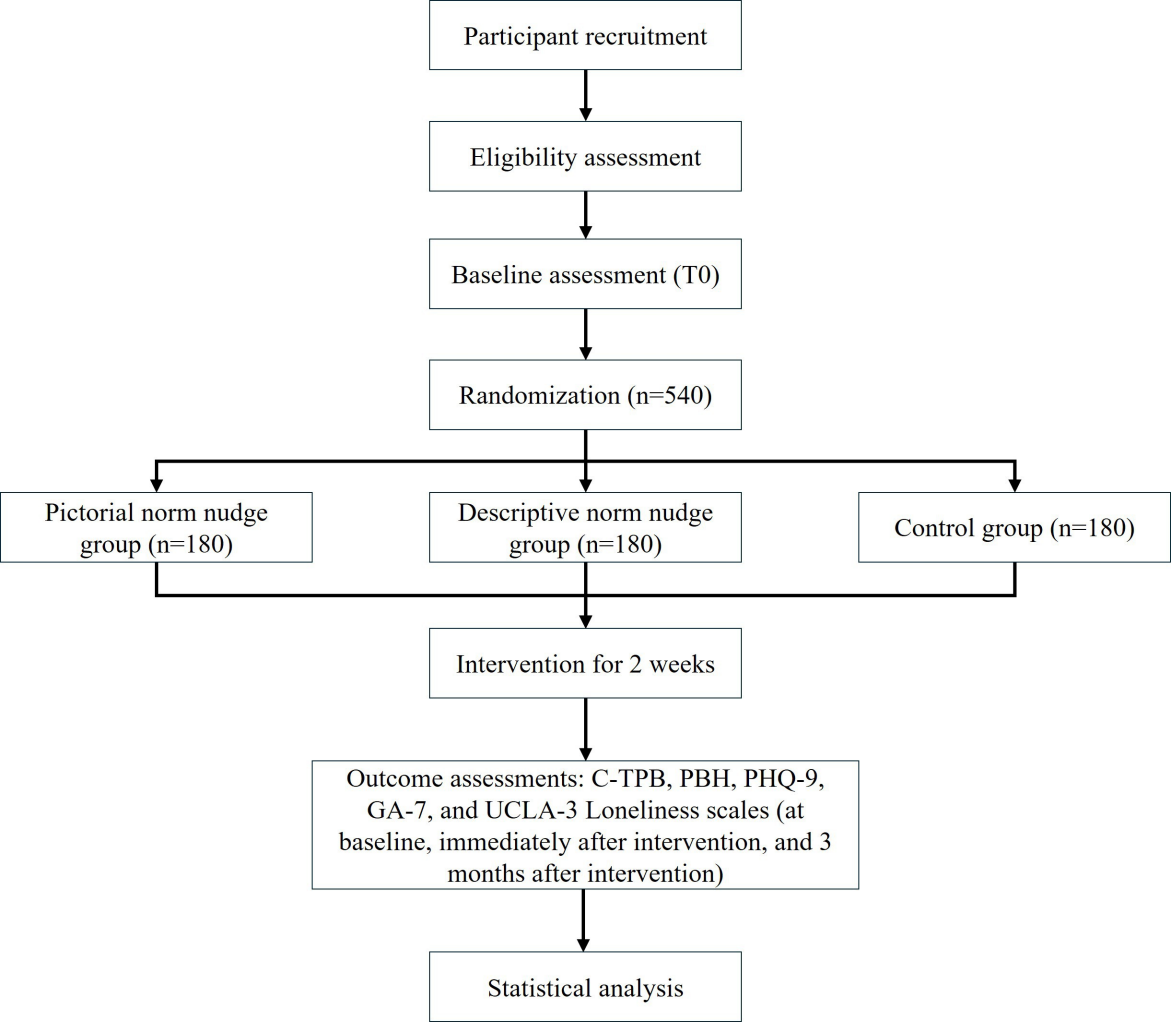
Flowchart of the trial design. C-TPB: Chinese version of the theory of planned behavior questionnaire; GAD-7: Generalized Anxiety Disorder–7 items; PBH: perceived barriers to help-seeking; PHQ-9: Patient Health Questionnaire–9 items; UCLA-3: University of California, Los Angeles–3 items.

A flowchart of the procedure is shown in Figure 1, and the study timeline is presented in Table 2. First, eligible individuals provide written informed consent either online or in person during the recruitment phase. Following enrollment, participants complete the baseline assessment (T0), which captures demographic information and baseline outcome measures. Randomization occurs immediately after baseline assessment. Participants then enter the 14-day intervention phase, during which they receive automated daily messages based on their assigned study arm. Immediately following this 14-day period, the postintervention assessment (T1) is administered. Finally, a follow-up assessment (T2) is conducted 3 months after the intervention to evaluate the sustainability of intervention effects. Completion of assessments at each time point is acknowledged with small tokens of appreciation to encourage retention.

**Table 2.** Schedule of enrollment, interventions, and assessments.

Study components	Enrollment	Allocation	Baseline assessment	Treatment (2 weeks)		Postintervention assessment	Follow-up assessment (3 months after the intervention)
				Week 1	Week 2		
Enrollment							
Eligibility screen	✓						
Informed consent	✓						
Allocation		✓					
Interventions							
Pictorial norm nudges group				✓	✓		
Descriptive norm nudges group				✓	✓		
Control group				✓	✓		
Assessments							
Demographic characteristics			✓				
Primary outcomes							
C-TPB^a^: subjective norms			✓			✓	✓
C-TPB: help-seeking intentions			✓			✓	✓
Secondary outcomes							
C-TPB: perceived behavioral control			✓			✓	✓
C-TPB: help-seeking attitudes			✓			✓	✓
Perceived barriers to help-seeking			✓			✓	✓
Patient Health Questionnaire–9 items: depressive symptoms			✓			✓	✓
Generalized Anxiety Disorder Scale–7 items: anxiety symptoms			✓			✓	✓
University of California, Los Angeles–3 items Loneliness Scale			✓			✓	✓

a C-TPB: Chinese version of the theory of planned behavior questionnaire.

### Assessment Plan

#### Outcome Measures

The primary outcomes for this trial are subjective norms and help-seeking intentions. Subjective norms regarding mental health help-seeking are measured using the 3-item subscale from the validated Chinese version of the TPB questionnaire (C-TPB), with sample items such as “Most people who are important to me think I should seek mental health services,” rated on a 6-point Likert scale (1=“do not agree at all” to 6=“very much agree”). Help-seeking intentions are similarly measured using the 3-item C-TPB intention subscale [15], with sample statements such as “I intend to seek mental health service.” Higher scores indicate greater levels of subjective norms and help-seeking intentions.

Secondary outcomes encompass perceived behavioral control and attitudes, measured using their respective 3-item and 6-item C-TPB subscales [15]. Sample questions include “I think I can decide whether to seek mental health or not” and “I think mental health services are useful,” rated on a similar 6-point Likert scale. Additionally, perceived barriers to help-seeking (PBH) are assessed using a 6-item scale adapted from the Chinese American Psychiatric Epidemiological Study [27]. Sample statements include “I do not know how to seek professional mental health help.” Responses are rated on a similar 6-point Likert scale as the C-TPB, with higher scores indicating greater PBH. Mental health conditions are thoroughly evaluated using the Patient Health Questionnaire–9 items (PHQ-9) for depressive symptoms [28], the Generalized Anxiety Disorder-7 (GAD-7) scale for anxiety symptoms [29], and the University of California, Los Angeles (UCLA) 3-item Loneliness Scale for feelings of isolation [30].

#### Demographic Variables

Age, gender, living status, education, years of employment, and self-rated physical health are collected at baseline.

#### Sample Size and Statistical Analyses

A power analysis for a 3-arm repeated-measures design indicates that 432 participants provide 80% power to detect a small effect size (d=0.15) at an α level of .05 using a linear mixed model. Accounting for an estimated 20% attrition rate across the study timeline, the target sample size is set at 540 participants.

Statistical analyses will follow the CONSORT (Consolidated Standards of Reporting Trials) guidelines using intention-to-treat (ITT) principles. To test the effectiveness of the interventions, a 2-level linear mixed-effects model will be used, with repeated measures (time points T0, T1, and T2) nested within individuals. The model will include fixed effects for group allocation, time, and the group-by-time interaction, along with a random intercept for participants to account for within-subject correlations.

Our primary planned contrasts are comparisons between the pictorial norm nudge and the active control groups, and between the descriptive norm nudge and the active control groups. The secondary comparison is between the pictorial norm nudge and the descriptive norm nudge groups. To address multiplicity issues arising from evaluating 2 primary outcomes (subjective norms and help-seeking intentions), a Bonferroni correction will be applied, setting the threshold for statistical significance for the primary outcomes at *P*<.025. Missing data will be handled using multiple imputation. Additionally, structural equation modeling using the baseline data will be used to compare the revised TPB pathway against the original theoretical model.

### Data Monitoring

The study procedures pose a low risk to participants. Participants’ well-being is monitored remotely throughout the trial, with established referral protocols in place should any participant experience distress. Trial progress and safety are reviewed in biweekly meetings led by the principal investigator.

### Patient and Public Involvement

Twelve community-dwelling older adults co-designed the nudge content but were not involved in the trial’s implementation. A journal manuscript will be prepared to present the results after the trial is completed; participants and partner community centers will be acknowledged in the manuscript. A brief summary of the results in plain language will be provided to all participants and community partners.

## Results

The study grant was awarded in June 2023, and the trial was prospectively registered on ClinicalTrials.gov (NCT06707935) on November 27, 2024. Recruitment and baseline assessments commenced on December 1, 2024 and concluded on June 30, 2026, a total of 566 participants had been recruited and screened for eligibility. As of June 30, 2026, 532 eligible participants have completed the baseline assessment, of whom 470 (88.34%) have completed the postintervention assessment (T1) and 408 (76.69%) have completed the final 3-month follow-up assessment (T2). The high completion rate to date indicates excellent feasibility of the remote, low-intensity delivery format. ITT analyses of the full dataset are anticipated by October 2026, with full results expected to be submitted for publication in early 2027.

## Discussion

### Expected Findings

This protocol describes an RCT evaluating co-designed norm-based nudges to promote mental health help-seeking intentions among older adults. We expect that modifying perceived subjective norms will directly improve help-seeking intentions, confirming the applicability of a culturally adapted TPB framework. Furthermore, by isolating textual and pictorial components, this study aims to clarify how visual communication formats influence the efficacy of digital nudges among older populations. If effective, the intervention will provide a scalable, low-cost mobile health (mHealth) strategy that can be readily integrated into community health promotion.

### Strengths and Limitations

A primary strength of this study is its participatory co-design approach, ensuring the nudges are culturally resonant and tailored to the target population’s lived experiences. Second, the rigorous 3-arm design isolates specific behavioral mechanisms by systematically controlling for informational and visual elements. Finally, the use of daily microinterventions via WhatsApp creates a highly scalable, low-burden model suited for real-world implementation.

However, certain limitations must be acknowledged. Outcomes rely on self-reported intentions rather than objective help-seeking behaviors, such as actual clinic visits, which may inflate the observed intervention effects. Additionally, the requirement for smartphone ownership introduces potential selection bias toward more digitally literate older adults, thereby slightly reducing the generalizability of the findings. Finally, the 3-month follow-up limits conclusions regarding long-term behavioral maintenance.

### Implications and Future Directions

If effective, this intervention may offer a scalable, low-resource strategy for promoting mental health help-seeking among older adults. Future research should extend this framework by examining objective service use metrics to determine whether intentions translate into actual behavior. Adapting the intervention for clinical populations or different cultural contexts will further validate the generalizability of norm-based nudging. Additionally, integrating these digital nudges directly into existing health care infrastructures or community services could be explored to facilitate immediate transitions from intention to actionable care.

### Conclusions

This trial evaluates a novel, co-designed digital nudge intervention targeting mental health help-seeking among older Chinese adults. By combining behavioral theory, participatory design, and ubiquitous mobile messaging, this study will generate valuable evidence on how to leverage choice architecture and social norms to improve mental health care use among aging populations.

## Supplementary material

10.2196/103455 Checklist 1 SPIRIT 2025 checklist.

10.2196/103455 Peer Review Report 1 Peer review report from the General Research Fund, Research Grants Council, Hong Kong SAR (Ref: 17615823).

## Abbreviations

C-TPB: Chinese version of the theory of planned behavior
CONSORT: Consolidated Standards of Reporting Trials
GAD-7: Generalized Anxiety Disorder–7 items
ITT: intention-to-treat
mHealth: mobile health
PBH: perceived barriers to help-seeking
PHQ-9: Patient Health Questionnaire–9 items
RCT: randomized controlled trial
SPIRIT: Standard Protocol Items: Recommendations for Interventional Trials
TPB: theory of planned behavior
UCLA: University of California, Los Angeles
