# Supplementary material for: Using Co-Designed Norm-Based Nudges to Promote Mental Health Help-Seeking Intentions Among Older Adults: Protocol for a Randomized Controlled Trial
Source: JMIR Res Protoc. 2026 Jul 21;15:e103455. doi: 10.2196/103455 (PMC13387629; doi:10.2196/103455)
Supplement: Checklist 1 [file resprot-v15-e103455-s001.pdf]

SPIRIT 2025 checklist of items to address in a randomized trial protocol*

Section / Topic	No	SPIRIT 2025 checklist item description	Reported on page no.
**Administrative information**			
Title and structured summary	1a	Title stating the trial design, population, and interventions, with identification as a protocol	Yes, in the title
	1b	Structured summary of trial design and methods, including items from the World Health Organization Trial Registration Data Set	Yes, in the abstract
Protocol version	2	Version date and identifier	Yes, in the abstract
Roles and responsibilities	3a	Names, affiliations, and roles of protocol contributors	Yes, in the title page, and the dedicated Authors' Contributions section
	3b	Name and contact information for the trial sponsor	Yes, corresponding author
	3c	Role of trial sponsor and funders in design, conduct, analysis, and reporting of trial; including any authority over these activities	Yes, in the funding statement
	3d	Composition, roles, and responsibilities of the coordinating site, steering committee, endpoint adjudication committee, data management team, and other individuals or groups overseeing the trial, if applicable	Yes, in the data monitoring session
**Open science**			
Trial registration	4	Name of trial registry, identifying number (with URL), and date of registration. If not yet registered, name of intended registry	Yes.
Protocol and statistical analysis plan	5	Where the trial protocol and statistical analysis plan can be accessed	Yes, in clinicaltrials.gov
Data sharing	6	Where and how the individual de-identified participant data (including data dictionary), statistical code, and any other materials will be accessible	Yes, in the data availability statement
Funding and conflicts of interest	7a	Sources of funding and other support (e.g., supply of drugs)	Yes, in the funding statement
	7b	Financial and other conflicts of interest for principal investigators and steering committee members	Yes, declared no conflict.
Dissemination policy	8	Plans to communicate trial results to participants, healthcare professionals, the public, and other relevant groups (e.g., reporting in trial registry, plain language summary, publication)	Yes, in the public involvement section
**Introduction**			
Background and rationale	9a	Scientific background and rationale, including summary of relevant studies (published and unpublished) examining benefits and harms for each intervention	Yes, in the introduction - background

	9b	Explanation for choice of comparator	Yes, active control
Objectives	10	Specific objectives related to benefits and harms	Yes, in the introduction - objective
**Methods: Patient and public involvement, trial design**			
Patient and public involvement	11	Details of, or plans for, patient or public involvement in the design, conduct, and reporting of the trial	Yes, in the participant and public involvement section
Trial design	12	Description of trial design including type of trial (e.g., parallel group, crossover), allocation ratio, and framework (e.g., superiority, equivalence, non-inferiority, exploratory)	Yes, in the study design section
**Methods: Participants, interventions, and outcomes**			
Trial setting	13	Settings (e.g., community, hospital) and locations (e.g., countries, sites) where the trial will be conducted	Yes, study setting and participants
Eligibility criteria	14a	Eligibility criteria for participants	Yes, study setting and participants
	14b	If applicable, eligibility criteria for sites and for individuals who will deliver the interventions (e.g., surgeons, physiotherapists)	N.A
Intervention and comparator	15a	Intervention and comparator with sufficient details to allow replication including how, when, and by whom they will be administered. If relevant, where additional materials describing the intervention and comparator (e.g., intervention manual) can be accessed	Yes, Intervention Program (PNN, DNN, Control)
	15b	Criteria for discontinuing or modifying allocated intervention/comparator for a trial participant (e.g., drug dose change in response to harms, participant request, or improving/worsening disease)	Yes, in the Delivery Format and Participant Engagement
	15c	Strategies to improve adherence to intervention/comparator protocols, if applicable, and any procedures for monitoring adherence (e.g., drug tablet return, sessions attended)	Yes, Engagement strategy (responses, daily messaging)
	15d	Concomitant care that is permitted or prohibited during the trial	N.A
Outcomes	16	Primary and secondary outcomes, including the specific measurement variable (e.g., systolic blood pressure), analysis metric (e.g., change from baseline, final value, time to event), method of aggregation (e.g., median, proportion), and time point for each outcome	Yes, Assessment plan – outcome measures
Harms	17	How harms are defined and will be assessed (e.g., systematically, non-systematically)	Yes, in the ethics and monitoring section
Participant timeline	18	Time schedule of enrollment, interventions (including any run-ins and washouts), assessments, and visits for participants. A schematic diagram is highly recommended (see Figure)	Yes, Table 2

Sample size	19	How sample size was determined, including all assumptions supporting the sample size calculation	Yes, in the sample size section
Recruitment	20	Strategies for achieving adequate participant enrollment to reach target sample size	Yes, in the study setting and recruitment description
**Methods: Assignment of interventions**			
Randomization:			
Sequence generation	21a	Who will generate the random allocation sequence and the method used	Yes.
	21b	Type of randomization (simple or restricted) and details of any factors for stratification. To reduce predictability of a random sequence, other details of any planned restriction (e.g., blocking) should be provided in a separate document that is unavailable to those who enroll participants or assign interventions	Yes, random allocation (1:1:1, parallel)
Allocation concealment mechanism	22	Mechanism used to implement the random allocation sequence (e.g., central computer/telephone; sequentially numbered, opaque, sealed containers), describing any steps to conceal the sequence until interventions are assigned	Yes, by independent research staff
Implementation	23	Whether the personnel who will enroll and those who will assign participants to the interventions will have access to the random allocation sequence	Yes, random allocation section
Blinding	24a	Who will be blinded after assignment to interventions (e.g., participants, care providers, outcome assessors, data analysts)	Yes, participants, assessors, and analysts
	24b	If blinded, how blinding will be achieved and description of the similarity of interventions	Yes, in the intervention section
	24c	If blinded, circumstances under which unblinding is permissible, and procedure for revealing a participant's allocated intervention during the trial	N.A
**Methods: Data collection, management, and analysis**			
Data collection methods	25a	Plans for assessment and collection of trial data, including any related processes to promote data quality (e.g., duplicate measurements, training of assessors) and a description of trial instruments (e.g., questionnaires, laboratory tests) along with their reliability and validity, if known. Reference to where data collection forms can be accessed, if not in the protocol	Yes, Table 2, Figure 1, and assessment plan section
	25b	Plans to promote participant retention and complete follow-up, including list of any outcome data to be collected for participants who discontinue or deviate from intervention protocols	Yes, token of appreciation
Data management	26	Plans for data entry, coding, security, and storage, including any related processes to promote data quality (e.g., double data entry; range checks for data values). Reference to where details of data management procedures can be accessed, if not in the protocol	Yes, in data availability section
Statistical methods	27a	Statistical methods used to compare groups for primary and secondary outcomes, including harms	Yes, in statistical analysis section

	27b	Definition of who will be included in each analysis (e.g., all randomized participants), and in which group	Yes, ITT stated
	27c	How missing data will be handled in the analysis	Yes, multiple imputation
	27d	Methods for any additional analyses (e.g., subgroup and sensitivity analyses)	Yes, SEM and subgroup analyses
**Methods: Monitoring**			
Data monitoring committee	28a	Composition of data monitoring committee (DMC); summary of its role and reporting structure; statement of whether it is independent from the sponsor and funder; conflicts of interest and reference to where further details about its charter can be found, if not in the protocol. Alternatively, an explanation of why a DMC is not needed	Not formal DMC for the low-risk nature and PI oversight
	28b	Explanation of any interim analyses and stopping guidelines, including who will have access to these interim results and make the final decision to terminate the trial	Interim analysis is described in the results
Trial monitoring	29	Frequency and procedures for monitoring trial conduct. If there is no monitoring, give explanation	Yes, biweekly PI-led monitoring
**Ethics**			
Research ethics approval	30	Plans for seeking research ethics committee/institutional review board approval	Yes, approval obtained
Protocol amendments	31	Plans for communicating important protocol modifications to relevant parties	N.A
Consent or assent	32a	Who will obtain informed consent or assent from potential trial participants or authorized proxies, and how	Yes, written consent from participants
	32b	Additional consent provisions for collection and use of participant data and biological specimens in ancillary studies, if applicable	Yes, consent for publication
Confidentiality	33	How personal information about potential and enrolled participants will be collected, shared, and maintained in order to protect confidentiality before, during, and after the trial	Yes, data anonymity statement
Ancillary and post-trial care	34	Provisions, if any, for ancillary and post-trial care, and for compensation to those who suffer harm from trial participation	Yes, referral protocol for distress

*We strongly recommend reading this checklist in conjunction with the SPIRIT 2025 Explanation and Elaboration and the SPIRIT 2025 Expanded Checklist for important clarifications on all the items. We also recommend reading relevant SPIRIT extensions. See www.consort-spirit.org

Citation: Chan A-W, Boutron I, Hopewell S, Moher D, Schulz KF, et al. SPIRIT 2025 statement: updated guideline for protocols of randomised trials. BMJ 2025;389:e081477. https://dx.doi.org/10.1136/bmj-2024-081477

© 2025 Chan A-W et al. This is an Open Access article distributed under the terms of the Creative Commons Attribution License (https://creativecommons.org/licenses/by/4.0/), which permits unrestricted use, distribution, and reproduction in any medium, provided the original work is properly cited.
