# Supplementary material for: Using Co-Designed Norm-Based Nudges to Promote Mental Health Help-Seeking Intentions Among Older Adults: Protocol for a Randomized Controlled Trial
Source: JMIR Res Protoc. 2026 Jul 21;15:e103455. doi: 10.2196/103455 (PMC13387629; doi:10.2196/103455)
Supplement: Peer Review Report 1 [file resprot-v15-e103455-s002.pdf]

23-24 GRF application

Overall Comments

This initiative builds out of the COVID pandemic and its adverse effects on the mental health of older people, including those in Hong Kong. The objectives of this application are to improve the somewhat stigmatised help-seeking behavior of older people concerning mental health (and reduce future possibilities of increased suicide completions evidenced in 2022 over earlier years) as well as to test the nudge theory compared to the older theory of planned behavior. This is an impressive proposal addressing an important topic, encouraging mental health support in older adults. The background and need for the project is well-established in this proposal and coheres with current literature.

Strengths:

The project's research questions and hypotheses are clearly stated, reflect the study's overall purpose, and are suitably addressed in the research agenda.

The project also has been piloted, with findings suggesting the importance of the research as proposed.

Tasks are identified and plotted on a monthly basis in a Gantt Chart for its duration.

The proposed budget is reasonable. It is consistent with the project aims, and each item with the budget is adequately justified.

The PI and Co-I have a strong record of academic and research accomplishment.

The co-design of the intervention is a real positive (even though a systematic review is still needed).

The PI and her team have constructed a research project that is original.

Weaknesses:

It would be helpful to have a bit more detail around the process to be used for the "iterative rounds of discussions with older volunteers and mental health professionals to refine the nudges" and how the pilot group of "30 community-dwelling older adults to test the feasibility of the social norm-nudges" will be identified.

I appeared that there was evidence for an enhanced impact with pictures and text (Houts et al., 2006) but not for pictures alone. Why pursuing a picture-alone approach?

It would have been nice to see the inclusion of an advisory group (older people with appropriate experience and professionals/ researchers).

The issue of low self recognition and cohort norms around 'coping' and not making a 'fuss' are not especially well discussed or cited. Evidence on perceptions of the availability and relevance of mental health services for older citizens is not considered in terms of existing evidence.

Research designed to improve help-seeking amongst older people is important and this proposal is timely in that regard. The applicants drawn on both nudge theory and the theory of planned behaviour to propose

a study designed to determine which of two approaches best convey social norms to older people: pictures or text?

However, a number of aspects are unclear, and some suggest that the proposal is somewhat premature.

Unclear:
i) the term 'revised TPB' has only the briefest explanation - within the text of the proposal and in Figure 1, and seems only loosely connected to research questions 2-4.
ii) research question 4 is about help-seeking, but the data only relate to improvements in subjective norm and mental health seeking intention'. Follow up is short, but ongoing intention, in the absence of help-seeking data, is not persuasive. In the Pathways to Impact Statement the PI writes that 'Although this study does not study help-seeking behaviour as the primary outcome, help-seeking intention is a strong predictor of subsequent behaviour, and behaviour may be hard to capture and require a larger sample size beyond the scope of the current study'. That is as may be, but surely it is important to endeavour to get some handle on help-seeking per se?
iii) The proposed 'pilot test' in Phase 1 is not a pilot in any conventional sense. What exactly is its purpose? Where does it fit into the study as a whole i.e. what will inform it, and what will it inform?
iv) The references to the Pandemic appear to add little value.

Premature
a) The literature reviews (which should be systematic reviews) proposed should be completed prior to funding being sought for a trial. There are effectively three reviews proposed: one on help=seeking in older Chinese with mental health needs, one on intervention programmes for changing social norms and increasing help seeking and one on the utility of TPB and nudge theory for modifying social norms.
b) In my view, the development of materials should be described in more detail, and probably at least commenced between the completion of the literature review and seeking funding for Phase 2 of the study. In fact, it seems from Figure 2 work appears already to be underway in this regard?

In addition, there is very little detail provided on the methods and procedures to be followed in the trial, and no discussion of ethical issues.

Potential Research Impact Comment:

The most original aspect of the proposed research is the use of a rigorous randomized control trial design to examine the effectiveness of the nudging approach in changing mental health help-seeking interventions. The use of picture nudges to encourage help-seeking could be used with other groups including the wider public but also more specifically children and people with learning difficulties.

At least three quarters of the "Pathways to Impact Statement simply repeats aspects of the research proposal. Only in the last paragraph does the PI focus on 'practical considerations' but here too there is no attention paid to how these contributions are going to come about, or what the research team is planning to do to maximise the impact of the study, should the findings be in the desired direction. The PI writes:

"This study will have a direct impact on its participants who would be exposed to mental health help-seeking social nudges, or mental health help-seeking knowledge. In the short-term, this intervention may increase participants’ mental health knowledge and help-seeking intention; in the long-term, this intervention may increase the chances that the participants initiate mental health help-seeking behaviour when they face mental health challenges in the future. In addition, this study will produce a set of

descriptive and pictorial social norm-nudges with associated effects in changing older people’s mental health help-seeking social norm and intention. "

The question remains, how will it have any such direct impact? And even if the participants in this study are positively impacted to seek help for mental health problems, how will the outputs from the study impact more widely?

Reviewer 1

VIEW COMMENTS FROM EXTERNAL REVIEWER

Project Number : 17615823

Project Title : Is a picture worth a thousand words? A participatory approach to co-design descriptive and pictorial social norm nudges and a randomised controlled trial (RCT) to examine their effectiveness in improving mental health help-seeking intention

PI Name : Dr Liu, Tianyin

Section A : Detailed Comments

1. Please comment on the objective(s) of the proposal, and whether the research agenda adequately addresses the objective(s)?

Excellent	Very Good	Good	Fair	Poor
◉	○	○	○	○

Comments:

The project's research questions and hypotheses are clearly stated, reflect the study's overall purpose, and are suitably addressed in the research agenda. This initiative builds out of the COVID pandemic and its adverse effects on the mental health of older people, including those in Hong Kong, and is illustrated by the increase in suicides among this population. It is appropriately theory-based, with demonstrated understanding of the related literature base. The project also has been piloted, with findings suggesting the importance of the research as proposed. This aspect of the proposal is further noteworthy in its backing, detail, and relevance. Theoretical considerations are nuanced and hypotheses well-stated. Finally, the need for such inquiry is ably demonstrated.

2. Please comment on the Research Design and Methodology.

Excellent	Very Good	Good	Fair	Poor
◉	○	○	○	○

Comments:

This multi-phased research design is well-described and consistent with the project's purpose and objectives. It includes written justification for each element. Indeed, rarely have I seen so much sound rationale and thorough discussion of methodology in a project proposal as I did here. That said, it would be helpful to have a bit more detail around the process to be used for the "iterative rounds of discussions with older volunteers and mental health professionals to refine the nudges" and how the pilot group of "30 community-dwelling older adults to test the feasibility of the social norm-nudges" will be identified (p. 25). Measurements are given, thoughtfully differentiated by primary or secondary outcomes. The data analysis plan is discussed in detail and consistent with the research aims.

3. Please comment on the feasibility of the proposed research.

Excellent	Very Good	Good	Fair	Poor
◉	○	○	○	○

Comments:

This is a proposed two-year project. Tasks are identified and plotted on a monthly basis in a Gantt Chart for its duration. Given the deep understanding of the project subject matter, clarity of research design and

methods, capacity of its PI and Co-PIs, and support from the University of Hong Kong, there is no reason to believe that the project, if funded, would not be achieved and within the time period described.

4. What do your consider to be the most original or innovative aspect of the proposed research? What advances would the research result bring about to the related field if the proposed research is successful?

Comments:

The most original aspect of the proposed research is the use of a rigorous randomized control trial design to examine the effectiveness of the nudging approach in changing mental health help-seeking interventions. If successful, the research may produce a varied set of social norm-nudges able to change older people's mental health help-seeking social norm and intention. The later, given current and increasing concerns about the mental health of older adults, makes the project important both in its timing and meaning.

5. Please comment on the reasonableness of the proposed budget and manpower planning and project duration.

Comments:

The proposed budget is reasonable. It is consistent with the project aims, and each item with the budget is adequately justified. The same applies to manpower planning and project duration. On the other hand, the education plan is somewhat general, and the letters of commitment are rather routine. The PI will be spending 15 hours weekly on the project, which is suggestive of substantial commitment to it and real investment of time. The Co-PIs only commit to 1-2 hours weekly, much more consultative in the giving of expertise and advice. There has been collaboration among all of them in the past, which is beneficial moving forward with this undertaking. Each also has a strong record of academic and research accomplishment.

6. Overall Comments

Overall Comment: This is an important project, with timely relevance, solid foundation, clear intent, sound methodology, and able personnel.

Strength: The grant application is strong throughout, and every major element is detailed and justified, addressing a problem that is both timely and critical.
Weaknesses: There are no major weaknesses.
Suggested improvements: A very few have been noted in other sections of this review.

Section B : Summary of Assessment

The project :

**Scientific/scholarly merit**	**Excellent**	**Very Good**	**Good**	**Fair**	**Poor**
	◉	○	○	○	○
**Duration Proposed**	**Too Long**	**Appropriate**	**Too Short**		
	○	◉	○		
**Impact of Research**	**High**	**Moderate**	**Low**	**None**	
	○	◉	○	○	

The principal investigator :

**Ability to undertake the proposal**	**Excellent**	**Very Good**	**Good**	**Fair**	**Poor**
	◉	○	○	○	○
**Track record in field**	**Excellent**	**Very Good**	**Good**	**Fair**	**Poor**
	◉	○	○	○	○

Reviewer 2

Section A : Detailed Comments

1. Please comment on the objective(s) of the proposal, and whether the research agenda adequately addresses the objective(s)?

Excellent	Very Good	Good	Fair	Poor
○	◉	○	○	○

Comments:

The overall project is ambitious as are the five objectives which are a mixture of practical and theoretical. The objectives are clearly described, and the overall approach of the project should allow most of these objectives to be realised. However, it was unclear to me how Objective 1 (investigating the application of the participatory approach) was to be achieved.

2. Please comment on the Research Design and Methodology.

Excellent	Very Good	Good	Fair	Poor
○	○	◉	○	○

Comments:

The proposed study is a theoretically based, three-arm RCT (with the control group receiving education only). The data analysis is well described, and the approach overall seems appropriate but I had a couple of queries. For example, reading the background section, it appeared that there was evidence for an enhanced impact with pictures and text (Houts et al., 2006) but not for pictures alone. Should this variation not be included in the trial? Also, I struggled to see how social norms were to be successfully depicted in picture form alone (I personally did not find the provided example provided convincing). The 'pilot' already undertaken by the applicants was not really a pilot - it did not really focus on the intervention and surprisingly included young people as participants without explanation.

3. Please comment on the feasibility of the proposed research.

Excellent	Very Good	Good	Fair	Poor
○	◉	○	○	○

Comments:

The team, who have worked together previously, have a good mixture of research backgrounds and are well placed to undertake the project. They have a wealth of research experience with older people. The fact that the proposed intervention can be 'piggy backed' on existing services is great and bodes well for successful recruitment of older participants.

4. What do your consider to be the most original or innovative aspect of the proposed research? What advances would the research result bring about to the related field if the proposed research is successful?

Comments:

The participatory approach, although increasingly common in the West, is relatively rare in Hong Kong and cold add to the methodological literature. I would imagine that if successful, the use of picture nudges to encourage help-seeking could be used with other groups including the wider public but also more specifically children and people with learning difficulties.

5. Please comment on the reasonableness of the proposed budget and manpower planning and project duration.

Comments:

The budget is reasonable for a project of this length and given the nature of their proposed role, it was sensible to request funds for a more expensive, suitably experienced RA. It was also good to see that 'thank yous' for participants were budgeted for. There were a couple of inconsistencies in the application. For example, early on the applicants say they plan two academic papers but in the budget they say they plan three papers. This needs clarifying. Similarly, the number of planned conference attendances needs clarification as this also seems inconsistent.
I also could not find a budget to pay the graphic designer. I imagine this is an omission.
The team is quite large (6 researchers) but some of them are expected to devote very little time to the project. However, this is probably sufficient as the PI plans to spend 15 hours per week on it. This seems realistic given the nature of the project.
However, looking at the Gantt chart, I was surprised how long was being allowed for dissemination - five months seems to be being planned for dissemination alone. This should not be necessary suggesting that perhaps the applicants do not need the full 24 months.

6. Overall Comments

Overall Comment: This is a very interesting ambitious project with a strong theoretical basis. The multi-disciplinary team are at a variety of points in their careers with good possibilities for researcher development with mentoring from the more senior researchers. The team have done some useful groundwork (although they rather optimistically refer to this as a 'pilot').

Strength: This is an important topic and the planned exploration of the theories behind the proposed intervention is good to see. The team is strong and the rationale for the planned approach is well argued. The background section is comprehensive and sets the scene well. The co-design of the intervention is also a real positive and the description of the planned data analysis is very clear. The focus on Chinese people is also well justified.

Weaknesses: However, at other times the application was rather unclear. I was slightly confused by the repeated references to the impact of the pandemic. Obviously this had, and still has, an impact in terms of social isolation and mental health but it did not really add to the rationale for the study. In my opinion, there is a strong enough rationale for the study without this emphasis on the pandemic.
Overall, I wondered whether the project is ready for funding at this point. Preparatory work is still needed and I was disappointed to see that as part of the proposal, the team plan to undertake a systematic review on which to base the co-design element of the study. I think this should have already been done.

Suggested improvements: I would have liked to see the inclusion of an advisory group (older people with appropriate experience and professionals/ researchers). It was particularly disappointing this was not included in the proposal given the participatory approach and co-design of the intervention. An advisory group should be involved with the project throughout. Even at the planning stage where they can contribute to the design and later they can add significantly to interpreting the findings and dissemination, especially locally.
I'd like to see more preparatory work including a systematic review. Ideally, I would have liked to see a proper pilot of the intervention but I appreciate that the participatory nature of the intervention development makes this difficult. A few more details such as why 14 days was selected for the provision of the 'nudges' would have been helpful and e.g., the text in Fig 2 needs revising. Finally, I was unable to find an impact statement in the application.

Section B : Summary of Assessment

The project :

**Scientific/scholarly merit**	**Excellent**	**Very Good**	**Good**	**Fair**	**Poor**
	○	◉	○	○	○
**Duration Proposed**	**Too Long**	**Appropriate**	**Too Short**		
	◉	○	○		
**Impact of Research**	**High**	**Moderate**	**Low**	**None**	
	○	◉	○	○	

The principal investigator :

**Ability to undertake the proposal**	**Excellent**	**Very Good**	**Good**	**Fair**	**Poor**
	○	◉	○	○	○
**Track record in field**	**Excellent**	**Very Good**	**Good**	**Fair**	**Poor**
	○	○	◉	○	○

Reviewer 3

Section A : Detailed Comments

1. Please comment on the objective(s) of the proposal, and whether the research agenda adequately addresses the objective(s)?

Excellent	Very Good	Good	Fair	Poor
◉	○	○	○	○

Comments:

The objectives of this application are to improve the somewhat stigmatized help-seeking behavior of older people concerning mental health (and reduce future possibilities of increased suicide completions evidenced in 2022 over earlier years) as well as to test the nudge theory compared to the older theory of planned behavior.

A randomized RCT model will compare text-based descriptive nudges and pictorial nudges with a knowledge-based component.
Phase 1 of the study will use ten older volunteers who have become mental health ambassadors to co-design the nudges with representatives from other social health fields as well as a graphic artist. Phase 2 will consist of a randomized trial comparing a descriptive text-based nudge with a pictorial image nudge along with a traditional control group.

This study addresses an important area with regard to older people.

2. Please comment on the Research Design and Methodology.

Excellent	Very Good	Good	Fair	Poor
◉	○	○	○	○

Comments:

The research design is solid. Older adults are included in the panel creating the "nudges" which is best practice in health -related research. The recruitment method, inclusion/exclusion criteria, and methods of analysis as well as opportunities for feedback loops are all fully addressed in this comprehensive proposal. A pilot test is also part of the design. Overall, the detailing of these methods provide great confidence in the ability of the researchers to complete this study.

3. Please comment on the feasibility of the proposed research.

Excellent	Very Good	Good	Fair	Poor
◉	○	○	○	○

Comments:

This study builds on the work of an ongoing JoyAge study. Many aspects of the proposed study are thus already available, such as recruitment group. Researchers are noted to be blind to which group they conduct telephone interviews with, which should also be apparent in the way questions are presented. All of the outcome assessment tools are clearly specified and relate to the objectives for the study in what is an admirably tight design overall.

4. What do your consider to be the most original or innovative aspect of the proposed research? What advances would the research result bring about to the related field if the proposed research is successful?

Comments:

The inclusion of Smartphone and apps with older people who are likely to be more comfortable with this approach over time as cohorts age up into older ages is important. The use of nudges to influence help-seeking for mental health which is somewhat stigmatised is novel. Further, there is a suggestion that this may be more effective in a cultural group that relies on external approval processes in a culture with a collectivist orientation, such as Hong Kong.

5. Please comment on the reasonableness of the proposed budget and manpower planning and project duration.

Comments:

The proposed budget seems reasonable. My only comment about manpower is that Terry Lum and Gloria Wong may only provide 1-2 hours per month on the project, equivalent of at most, 24 hours or 3 days per year. That seems a very small amount of time to both be brought up to date on the project operation and to also provide benefit of their expertise at multiple stages. Their involvement is part of the reason for high confidence in the completion and outcomes of this project.

6. Overall Comments

Overall Comment : This is an impressive proposal addressing an important topic, encouraging mental health support in older adults. The background and need for the project is well-established in this proposal and coheres with current literature. The methods are discussed in a detailed manner and promise to provide information about the desired outcomes, meeting the objectives.. Further it is tightly and even elegantly written, addressing the areas about which I would typically have questions.

Strength: This is a strong proposal.

Weaknesses: None noted.

Suggested improvements: The only minor point would be to see more involvement by two key researchers listed on this study, if their time would allow.

Section B : Summary of Assessment

The project :

**Scientific/scholarly merit**	**Excellent**	**Very Good**	**Good**	**Fair**	**Poor**
	◉	○	○	○	○
**Duration Proposed**	**Too Long**	**Appropriate**	**Too Short**		
	○	◉	○		
**Impact of Research**	**High**	**Moderate**	**Low**	**None**	
	◉	○	○	○	

The principal investigator :

**Ability to undertake the proposal**	**Excellent**	**Very Good**	**Good**	**Fair**	**Poor**
	(•)	( )	( )	( )	( )
**Track record in field**	**Excellent**	**Very Good**	**Good**	**Fair**	**Poor**
	( )	(•)	( )	( )	( )

Reviewer 4

Section A : Detailed Comments

1. Please comment on the objective(s) of the proposal, and whether the research agenda adequately addresses the objective(s)?

Excellent	Very Good	Good	Fair	Poor
○	◉	○	○	○

Comments:

Older people and mental health is a Cinderella subject in social welfare. This is mainly because attention in the social welfare field is usually focused on younger people as they are the 'next generation' and are deemed to warrant particular support and intervention. This proposal has been submitted by an experienced PI who has put together an accomplished team to execute the research should the proposal be successful. The objective of improving mental-health help-seeking intention is a worthy and important one and the research agenda adequately addresses this objective.

2. Please comment on the Research Design and Methodology.

Excellent	Very Good	Good	Fair	Poor
◉	○	○	○	○

Comments:

The research design and methodology is relatively straight forward with an initial participatory approach with professionals followed by three-arm RCT with 540 older adults. This element of the research comprises of a control group and an intervention group. I particularly like the fact the researchers will be blind to group allocation so ensuring an objective approach is sought.

3. Please comment on the feasibility of the proposed research.

Excellent	Very Good	Good	Fair	Poor
○	◉	○	○	○

Comments:

As indicated above this is a straight forward piece of research which has a competent and experienced team working to deliver a successful project. Further, the involvement of a Co-I with a working link with a relevant NGO must give confidence that the research will achieve its stated goals.

4. What do your consider to be the most original or innovative aspect of the proposed research? What advances would the research result bring about to the related field if the proposed research is successful?

Comments:

The PI and her team have constructed a research project that is original. Mental health and older people is an under researched aspect of social welfare. This together with examining how pictorial social norm nudges can be more successful than knowledge based text messages in changing and improving mental health is innovative and could have implications for practice with older people.

5. Please comment on the reasonableness of the proposed budget and manpower planning and project duration.

Comments:

The proposed budget and the manpower planning is appropriate and the project duration of this sort of research is adequate.

6. Overall Comments

Overall Comment : This is an interesting and important piece of research that the RGC might like to seriously consider for funding. The results of the work could have implications for the delivery of effective work with older people in Hong Kong.

Strength: Innovative and original research that will be delivered by a strong research team

Weaknesses: 540 older adults need to be recruited for the main part of the field work. This is a significant number. What would happen if the researchers had to deal with a smaller group of respondents?.

Suggested improvements: A key to the success of this work will be the relationship between the academic institution and the NGO. It will be important therefore to ensure this relationship is nurtured and fostered.

Section B : Summary of Assessment

The project :

**Scientific/scholarly merit**	**Excellent**	**Very Good**	**Good**	**Fair**	**Poor**
	○	◉	○	○	○
**Duration Proposed**	**Too Long**	**Appropriate**	**Too Short**		
	○	◉	○		
**Impact of Research**	**High**	**Moderate**	**Low**	**None**	
	◉	○	○	○	

The principal investigator :

**Ability to undertake the proposal**	**Excellent**	**Very Good**	**Good**	**Fair**	**Poor**
	◉	○	○	○	○
**Track record in field**	**Excellent**	**Very Good**	**Good**	**Fair**	**Poor**
	◉	○	○	○	○

Reviewer 5

Section A : Detailed Comments

1. Please comment on the objective(s) of the proposal, and whether the research agenda adequately addresses the objective(s)?

Excellent	Very Good	Good	Fair	Poor
○	◉	○	○	○

Comments:

This is a potentially relevant study which sets out to examine the value of nudge theory focusing on the 'social norm' element of TBP theory. The proposal sets a relevant context for the study which includes the lack of investigation of the potential value of social norm nudges in mental health promotion and help seeking. The challenges of timely help seeking amongst older citizens is evidenced. The issue of low self recognition and cohort norms around 'coping' and not making a 'fuss' are not especially well discussed or cited. Evidence on perceptions of the availability and relevance of mental health services for older citizens is not considered in terms of existing evidence.
I liked the co-production element of the study (phase one). It is useful to encourage older citizens and other stakeholders to actively participate in the development of social norm nudges and the visual / pictorial elements proposed for arm one of the subsequent RCT. The RCT is reasonably justified and access to a potential sample does not seem to pose any major problems. The breadth of measurements used in BL/post and follow up is significant - it would be helpful to know precisely which measures and combination of measures are intended to address which aspect of the research; time commitment and researcher awareness of the potential for participant burden.

2. Please comment on the Research Design and Methodology.

Excellent	Very Good	Good	Fair	Poor
○	◉	○	○	○

Comments:

The approach seems reasoned. See above re: range of proposed measures x 3 Time frames.

3. Please comment on the feasibility of the proposed research.

Excellent	Very Good	Good	Fair	Poor
○	◉	○	○	○

Comments:

Existing relationships with services seems to adequately address the issue of accessing potential participants. I would have liked to have seen more information on the timing / time allowed for co production as this element of research is often significantly under estimated.

4. What do your consider to be the most original or innovative aspect of the proposed research? What advances would the research result bring about to the related field if the proposed research is successful?

Comments:

Social norm nudge theory and the use of pictorial elements to potentially encourage mental health help seeking is novel. To what extent has research considered / evidenced the acceptability of current mental health services for older people?

5. Please comment on the reasonableness of the proposed budget and manpower planning and project duration.

Comments:

Budget is reasonable. Team are well constructed and a good mix of experienced and developing career researchers.

6. Overall Comments

Overall Comment :

Has the potential to be an interesting study.

Strength:

Social nudge theory and the use of pictorial cues.

Weaknesses:
Context could have been improved including take up / attitudes / availability, acceptability and accessibility of existing mental health services.
Time allowed for co production - and approach could have been a little more developed
Impact / approach to impact could have been further developed

Suggested improvements:
As above

Section B : Summary of Assessment

The project :

**Scientific/scholarly merit**	**Excellent**	**Very Good**	**Good**	**Fair**	**Poor**
	○	○	◉	○	○
**Duration Proposed**	**Too Long**	**Appropriate**	**Too Short**		
	○	◉	○		
**Impact of Research**	**High**	**Moderate**	**Low**	**None**	
	○	◉	○	○	

The principal investigator :

**Ability to undertake the proposal**	**Excellent**	**Very Good**	**Good**	**Fair**	**Poor**
	○	◉	○	○	○
	**Excellent**	**Very Good**	**Good**	**Fair**	**Poor**

**Track record in field**	○	◉	○	○	○
